# Anthropometric and Motor Profile of Table Tennis Players: A Systematic Literature Review

**DOI:** 10.3390/jfmk11030334

**Published:** 2026-08-26

**Authors:** Alessandro Guarnieri, Valentina Presta, Fabiana Laurenti, Salvatore Mazzei, Giuliana Gobbi, Giancarlo Condello

**Affiliations:** 1 Department of Medicine and Surgery, University of Parma, Via Gramsci 14, 43126 Parma, Italy; alessandro.guarnieri@unipr.it (A.G.); valentina.presta@unipr.it (V.P.); fabiana.laurenti@unipr.it (F.L.); salvatore.mazzei@unipr.it (S.M.); 2 Department of Neurosciences, Biomedicine and Movement Sciences, University of Verona, Piazzale Ludovico Antonio Scuro 10, 37124 Verona, Italy

**Keywords:** racket sports, somatotype, body composition, strength, change-of-direction, reaction time

## Abstract

**Background**: While the biomechanical, physiological, and nutritional determinants of table tennis (TT) performance have already been summarized, a dedicated synthesis of players’ anthropometric and motor characteristics is still lacking. This review aimed to synthesize current evidence across different ages, genders, and competitive levels. **Methods**: BASE, PubMed, ScienceDirect, Scopus, Semantic Scholar, and Web of Science databases were searched for study selection. Cross-sectional studies investigating the anthropometric and motor characteristics of healthy TT players aged 7–40 years were considered eligible. **Results**: Thirty-one studies were included. Eighteen studies focused on motor characteristics, seven on anthropometric traits, and six examined both. **Conclusions**: Adult male TT players predominantly exhibit an endomorph–ectomorph profile with greater lean mass, whereas females show an endomorph–ectomorph somatotype. Moreover, higher-ranked athletes generally display greater lean mass. Compared to specific strength-based sports athletes, TT players tend to have lower limb circumferences and lower lean and muscle mass, while showing higher body fat than specific speed- and agility-based sports. Higher-level players tend to demonstrate faster reaction and movement times, as well as superior change-of-direction abilities. Compared to other racket sports, they tend to exhibit superior coincidence-anticipation timing under high stimulus velocities and distinctive motor coordination. In terms of strength, TT players show proficiency in horizontal jumps and dynamic back strength, despite lower vertical explosive power. The identified characteristics provide a descriptive profile of table tennis players and may inform future longitudinal research investigating their potential relevance for talent identification and development.

## 1. Introduction

Table tennis is a widely popular racket sport and counts more than 260 million players around the world, with approximately 33 million engaging in competitive play [1]. This sport is accessible to individuals of all ages and also offers a variety of health benefits for children’s development [2] and older adults’ preservation of physical and cognitive functioning [3,4,5]. Since its Olympic debut in 1988, the sport has garnered attention for its intense competitiveness and for the multifaceted physical demands requested to the players. In fact, to excel in competition, table tennis players need high levels of speed, strength, power, flexibility, and quick reactions, as a consequence of the dynamic nature of the game [6]. Both Olympic and Paralympic matches have been largely investigated in terms of strokes and footwork, for the determination of performance models [7,8,9,10,11,12]. However, the optimization of athletic performance requires the determination of a complete player profile to guide talent identification and development [13] and to monitor the effectiveness of training programs [14]. Recent reviews have summarized biomechanical [1,15,16], physiological [1,17], and nutritional [18] traits of table tennis players. The physical and anthropometric characteristics of table tennis players have also been included in reviews synthesizing a variety of sports [19,20,21], or more specifically racket sports, including also tennis [22], badminton [23], and squash [14]. Despite these efforts, a dedicated synthesis focused on the anthropometric and motor characteristics of table tennis players is still lacking.

Therefore, the primary aim of this study was to conduct a systematic literature review to provide a comprehensive synthesis of the anthropometric and motor characteristics of table tennis players, highlighting the differences among ages, gender, competitive levels, and different sports. The available evidence could be used to delineate a comprehensive anthropometric and motor profile for table tennis players. This profile is established by stratifying the data to highlight the distinguishing features of table tennis players relative to other athletes and detailing how these traits vary across different ages, genders, and competitive levels.

## 2. Materials and Methods

### 2.1. Protocol Registration

This systematic review has been officially registered in the International Prospective Register of Systematic Reviews (PROSPERO; CRD42023443064; July 2023).

### 2.2. Search Strategy

The literature search was performed in November 2023 by two independent reviewers for articles published between the years 2000 and 2023 across the databases PubMed, Scopus, and Web of Science, and the academic search platforms BASE, Semantic Scholar, and ScienceDirect, according to the Preferred Reporting Items for Systematic Reviews and Meta-Analyses [24]. The following keywords and Boolean operators were used as search terms: (anthropometry OR anthropometric OR somatic OR somatotype OR “body composition”) AND “table tennis”; (“motor task” OR “motor level” OR “motor performance” OR “motor fitness” OR “physical performance” OR “physical fitness” OR “physical level”) AND “table tennis”. The exact full search syntax adapted for each specific database and platform is reported in Supplementary Table S1. Additionally, to maximize sensitivity and capture specific motor-domain studies potentially missed by the primary search terms, the reference lists of selected articles and relevant reviews were manually screened (citation searching). A supplementary search was conducted to identify new eligible articles published between November 2023 and December 2025.

### 2.3. Study Selection and Eligibility Criteria

Studies assessing the anthropometric and/or motor characteristics of young and adult individuals involved in table tennis were initially screened by two independent reviewers based on title and abstract. Furthermore, studies were screened for meeting the following inclusion criteria: (i) cross-sectional study designs; (ii) individuals aged 7–40 years (age range specifically selected to include athletes eligible for official competitive tournaments and to exclude the “Master” category >40 years old); (iii) absence of physical or mental illness; (iv) published in peer-review journals; (v) availability of full text in English. In addition, studies investigating only cardiorespiratory, metabolic, or injury-related variables were excluded. To be classified as “table tennis players”, participants had to be actively involved in the sport (e.g., regular training, club affiliation, or participation in official competitions). For studies involving mixed-age samples extending beyond our age limit, the study was excluded unless data for the eligible age subgroups could be cleanly extracted. Duplicates were identified and removed using Microsoft Excel (v2608) spreadsheet functions. The screening process was performed entirely independently by two reviewers. Any disagreements between the two primary reviewers during the screening phase were resolved through discussion to reach a consensus; if consensus could not be reached, a third reviewer was consulted to make the final decision. Title, abstract, and full text were screened and assessed for eligibility using standard extraction forms, including study design, sample characteristics, and outcomes. A complete list of the excluded full-text articles, along with the specific reasons for their exclusion, is provided in Table S2 (Supplementary Table S2).

### 2.4. Data Extraction

The data extraction process was independently conducted by two different authors, with disagreement resolved by consensus; if consensus could not be reached, a third reviewer was consulted to make the final decision. The extracted data included: study design, sample characteristics (mean and range age, total sample size, gender distribution, subgroup sizes, training age), competitive levels, type of measurements (including reliability and validity), numerical group results and effect estimates with uncertainty (when reported), main findings, and study fundings/conflict of interest. For youth samples, the presence or absence of biological maturity assessments was also recorded. Only the primary study characteristics, measurement methodologies, and main findings have been summarized and presented in the final table, Supplementary Table S3.

### 2.5. Risk of Bias Assessment

The study quality and risk of bias of the included studies were independently evaluated by two authors using the Joanna Briggs Institute (JBI) critical appraisal tool for cross-sectional studies [25]. Each item in the tool required assessment through responses categorized as “yes”, “no”, “unclear”, or “not applicable”. Specifically, Item 3 (“Was the exposure measured in a valid and reliable way?”) was systematically rated as NA because, in the context of the included descriptive studies profiling anthropometric and motor traits, an epidemiological “exposure” cannot be defined. A scoring system was employed wherein a positive response (“yes”) received 1 point, while a negative response (“no”, “unclear”) received 0 points. NA items were excluded from the denominator when calculating the final percentage. The total scores were converted into percentages and classified as follows: “high” (>80%), “moderate” (50–79%), and “low” (<50%) quality.

### 2.6. Data Synthesis

The feasibility of a quantitative analysis (meta-analysis) was evaluated separately for each outcome. However, due to the high heterogeneity in methodological approaches, measurement protocols, and participant characteristics (age ranges, gender, competitive levels) across studies, statistical pooling was deemed inappropriate. Furthermore, some of the included studies only reported *p*-values and lacked effect sizes, confidence intervals, or complete descriptive statistics. Therefore, the data synthesis was performed according to the Synthesis without meta-analysis (SWiM) guidelines [26]. Since quantitative effect estimates could not be determined, the synthesis relied on structured effect direction. The included studies were grouped and analyzed based on their investigated primary outcomes (i.e., motor and/or anthropometric characteristics) and further sub-grouped by specific comparisons (i.e., age, gender, competitive levels, and sports). When multiple outcomes from the same study were reported, they were extracted and synthesized individually. For studies involving mixed age, competitive levels, or sport samples, data were considered only if subgroup results were reported separately for each subgroup. The narrative synthesis explicitly incorporates the risk of bias assessment, giving greater interpretative weight to findings derived from moderate-to-high quality studies.

## 3. Results

### 3.1. Selection Process

**Figure 1:**
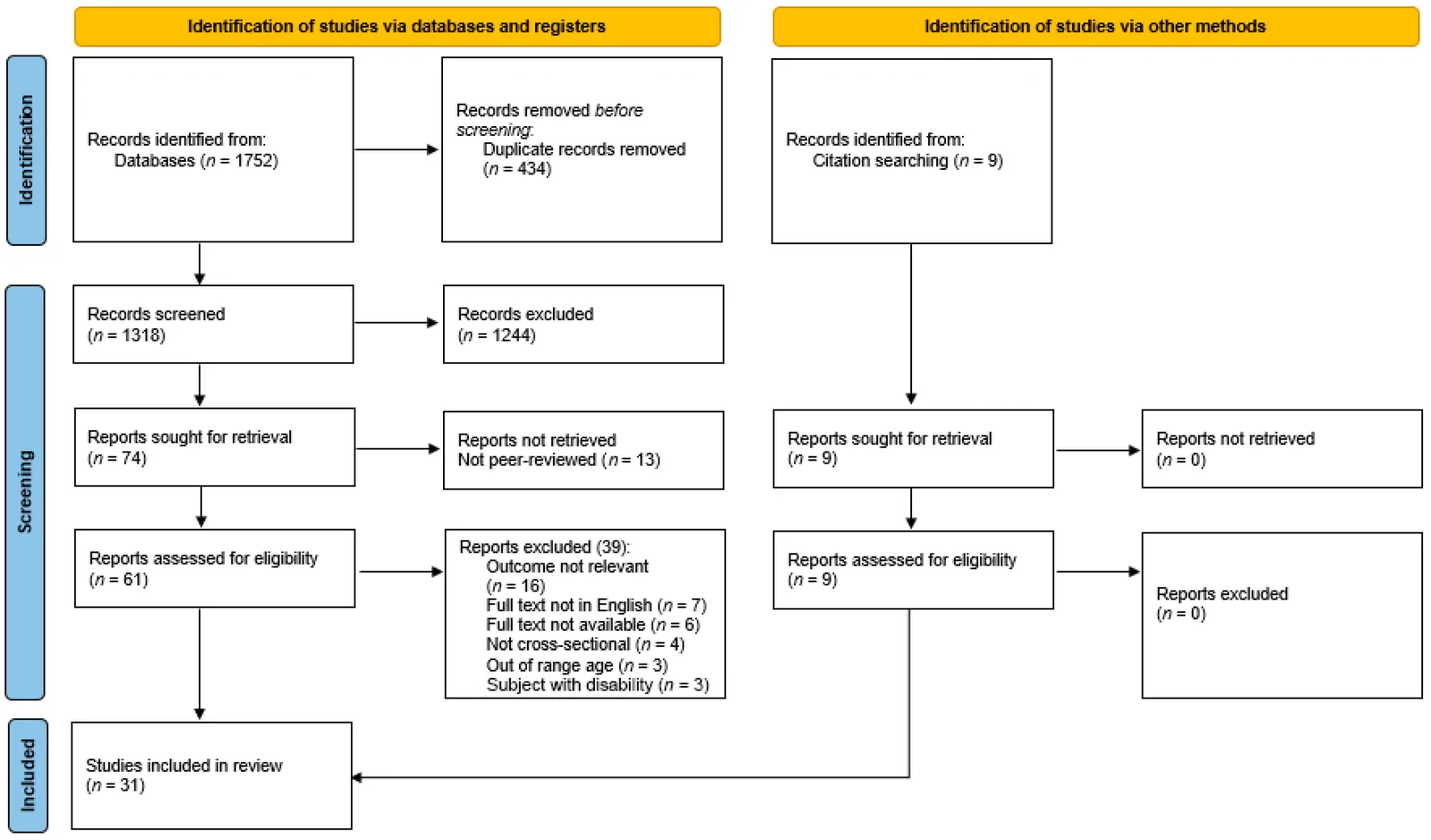
Preferred Reporting Items for Systematic Reviews and Meta-Analyses (PRISMA) flow diagram of articles selected and included in the systematic review.

A total of 1752 studies were initially identified. After the removal of 434 duplicates, 1318 unique studies were screened by title and abstract. Seventy-four articles were selected for screening against the eligibility criteria, of which 13 were not retrieved. Sixty-one full-text article reports were assessed for eligibility. Following the evaluation, 39 articles were excluded for not meeting the inclusion criteria. Four articles initially appeared to meet the inclusion criteria but were excluded due to differences in outcomes [27,28,29,30]. The complete list of excluded reports and reasons is detailed in Supplementary Table S2. Nine additional studies were included through citation searching. A final number of 31 studies were included in this systematic review (Figure 1).

### 3.2. Study Characteristics

Table S3 of the Supplementary Materials (Supplementary Table S3) summarized the characteristics of the included studies. Publication dates of the studies ranged between 2010 and 2025, and all of them adopted the cross-sectional study design. Spain emerged as the most investigated country (*n* = 9), followed by India (*n* = 7), Turkey (*n* = 4), China (*n* = 2), and Poland (*n* = 2). A single study was conducted in Belgium, Brazil, Italy, Korea, Saudi Arabia, Slovakia, and the Netherlands. The total sample size ranged from 16 to 2500 participants, while the specific table tennis subgroup sizes ranged from 12 to 495. Thirteen of the included studies considered young participants <18 years, 7 focused on adult individuals, and 5 investigated both youth and adult participants. Six studies did not report criteria for age sampling. Regarding gender, 15 of the included studies considered both male and female players, 13 studies investigated males, 1 focused on females, and 1 study did not report the sample gender. Considering the determination of the anthropometric and motor profile, 5 studies delved into age differences, 10 studies investigated gender differences, 4 studies explored differences among competitive levels, and 20 studies compared table tennis players with other athletes from different sports and non-athletes.

### 3.3. Investigated Outcomes

Among the included studies, 18 of them investigated motor outcomes [31,32,33,34,35,36,37,38,39,40,41,42,43,44,45,46,47,48], 7 studies explored anthropometric outcomes [49,50,51,52,53,54,55], and 6 studies considered both motor and anthropometric outcomes [56,57,58,59,60,61]. Measurements and main findings are summarized in Table S3.

#### 3.3.1. Assessment of Anthropometric Outcomes

The anthropometric outcomes were described using different measurement sets across studies. Most followed the International Society for the Advancement of Kinanthopometry (ISAK) [62] guidelines [53,56,58], assessing body mass, height, eight skinfold thicknesses (i.e., biceps brachii, triceps, subscapular, iliac crest, supraspinal, abdominal, thigh, and medial calf), four girths (i.e., biceps brachii relaxed and contractedflexed, thigh, and calf), and three breadths (i.e., biepicondylar femur, biepicondylar humerus, and bistiloyd wrist). Alternatively, one study [60] followed The Martin and Saller guidelines [63], including body mass, height, sitting height, arm span, five skinfold thicknesses (i.e., triceps, suprailiac, subscapular, medial calf skinfold, and calf), five girths (i.e., arm relaxed, waist, hip, thigh, and lower leg), and two breadths (i.e., humerus and femur). One study collected eight skinfolds (i.e., triceps, chest, midaxillary, abdominal, subscapular, suprailiac, thigh, calf), two girths (i.e., flexed bicep and calf), and two breadths (i.e., femur and humerus) [54]. A different set includes measurements such as body mass, height, sitting height, arm span, two skinfold thicknesses (i.e., abdomen and upper arm), three girths (i.e., chest, calf, and waist), three lengths (i.e., arm, leg, and lower leg), two widths (i.e., shoulder and crista), two circumferences (i.e., thigh and ankle), Achilles tendon length, and subscapular angle [61]. A further set focused on four skinfold thicknesses (i.e., triceps, forearm lateralis, forearm volaris, and mid-thigh) and four girths (i.e., maximal upper arm, maximal forearm, mid-thigh, and maximal calf) [51]. In one study, only body mass, height, and three skinfold thicknesses (i.e., triceps, subscapular, calf) were analyzed [57].

Body composition was evaluated using dual-energy X-ray absorptiometry (DXA) to measure fat-free mass (FFM), fat mass (FM), fat-free soft tissue mass (FFSTM), body fat percentage (%BF), and bone mineral density (BMD) and content (BMC). Bioelectrical impedance analysis (BIA) was used in two studies to estimate weight, fat content, fat-free mass, total water content, and body cell mass index (BCMI) [49,52]. Moreover, the sum of six skinfolds (i.e., triceps, subscapular, suprailiac, abdominal, thigh calf, and medial calf), upper body skinfolds (i.e., triceps and subscapular), trunk skinfolds (i.e., suprailiac and abdominal), and lower body skinfolds (i.e., thigh calf and medial calf) was applied in one study to calculate fat content [50]. In the same study [50], fat mass and percentage were calculated using the Yuhasz equation modified by Faulkner [64], bone mass and percentage using the Rocha method [65], and muscle mass and percentage according to Martin’s equation [66]. Additionally, a specific equation [67] for the estimation of fat mass, lean mass, and bone density was applied in 3 studies [53,54,56]. Furthermore, one study [58] followed the Durnin and Womersley technique [68] to examine body density, whilst body fat percentage was derived from Brozek’s equation [69]. Body density was calculated in one study [54] following the Jackson and Pollock formula [70]. In one study, the equations applied to estimate body fat and lean percentage were not reported [57].

Finally, the Hemysfield equation [71] was used to estimate cross-sectional area (CSA) for arm, leg, and thigh [56], while all the somatotype assessments [53,54,56,58,60] followed the Carter and Heath method [72].

#### 3.3.2. Assessment of Anthropometric Outcomes

Reaction time (RT) was evaluated employing different instruments and methodologies. Simple visual (VRT) and auditory (ART) reaction times were assessed using software- and sensory-based equipment requiring participants to press a button after the stimulus presentation [34,36,40,61]. Studies used reactive photoelectric systems to evaluate simple and choice VRT with different stimulus delays [39], and 5 m lateral sprints to test choice VRT and displacements [35,44]. Other VRT protocols required participants to press a switch in response to a green stimulus, or to choose between two stimuli in the choice reaction test [31,39]. Moreover, simple and choice VRT and simple ART were assessed by asking participants to jump off a reaction mat [57]. One study did not provide information about the ART test [32]. Coincidence-anticipation timing was assessed by measuring the accuracy of predicting moving lights’ arrival at varying velocities [31,33]. In one study, the experimental procedures for the assessment of VRT were not reported [47].

Explosive and elastic leg strength were commonly measured using squat jump (SJ) [42,45,46,48,58,61] and countermovement jump (CMJ) tests [42,43,44,45,48,58,59], respectively. Additionally, general vertical jumps [37,57], horizontal CMJ (HCMJ) [41,42], standing broad jump [38,47,59], and Abalakov jump [45] were administered. Drop jump (DJ) was employed to evaluate reactive strength [39]. Upper body strength was assessed using the handgrip test (HANDG) [41,44,45,47,53,58], knee push-ups test [59], sit-ups test [57,59], medicine ball throws [44,47], and power deadlifts [61].

Flexibility was commonly measured with the sit-and-reach (SAR) test [41,45,54,59]. Additionally, a shoulder rotation test was administered for further evaluation [59]. Flexibility was also evaluated across various body segments, including the lower back, hamstrings, ankles, trunk, neck, and shoulders, even though the testing procedures were not provided [58].

Change-of-direction (COD) ability was evaluated in several studies using the 505-agility test [39], modified agility test [41], and shuttle run [46]. Different movement patterns were also tested in a study, with a circuit consisting of climbing down and over various equipment [37]. Experimental procedures were not reported in two studies [47,58].

Speed was assessed using linear acceleration over the distance of 5 m [39,41,59], 20 m [39,47], and flying 30 m [45].

Gross motor coordination was examined with the “Körperkoordinations Test für Kinder” [73], including the backward balance test, the jumping sideways test, and the moving sideways test [59]. The same study [59] evaluated dribbling performance and overarm throwing skills through the UGent Dribbling Test [74] and the overhead-throwing test, respectively. The perceptuomotor skills assessment was applied in one study, consisting of speed evaluation while dribbling, aiming at a target, ball skills, throwing a ball, and eye–hand coordination tests [37].

For the evaluation of reaction and displacement speed during the game, the Table Tennis Specific Battery Test (TTSBT) [75] was administered [60]. The player has to perform forehand (T1) or backhand (T2) top-spins from different areas of the table tennis table. In T3, balls are thrown at higher speed to the sides of the table, and the player should alternately perform forehand and backhand top-spins.

### 3.4. Determination of the Anthropometric Profile

The outcomes evaluated in the included studies were compared according to age, gender, competitive level, and sport for the determination of the anthropometric profile of table tennis players.

#### 3.4.1. Age

Two studies explored differences among age groups in table tennis players for the anthropometric characteristics. Age-related increments in body weight, height, and sitting height, humerus and femur diameter, subscapular skinfold, arm span, and biceps, waist, hip, thigh, and calf girth were revealed between two groups of young players aged 9–12 and 13–17 years old [60]. Significant age effects were reported for height, weight, body mass index, arm girth (relaxed and tensed), CSA (arm, leg, thigh), bone development, and lean mass across five age groups (i.e., Under 11, Under 13, Under 15, Under 18, and Senior), even though no further post hoc comparisons were provided [56].

#### 3.4.2. Gender

Four studies investigated gender differences in table tennis players for the anthropometric characteristics. Morphological analysis revealed larger biepicondylar humerus and femur breadth [56,58], and greater biceps, calf, and waist girth [56] in males. In terms of body composition, males exhibited higher lean body mass compared to females [55,58]. Additionally, a single study highlighted a higher percentage of muscle mass in senior male players [56]. Conversely, female players showed higher fat body mass [55,58] and body fat percentage [55,56,58] than male players. For the somatotype examination, the endomorph–mesomorph somatotype was predominant in males, whilst the endomorph–ectomorph somatotype was prevalent in females [54,58,60].

#### 3.4.3. Competitive Level

Two studies compared the anthropometric characteristics among different competitive levels of table tennis players. When three distinct levels were categorized (i.e., international, national, regional), international male players exhibited lower fat-free mass and fat-free soft tissue mass in their dominant arm than national players, and in their non-dominant arm than both national and regional players [55]. Similarly, international female players showed higher bone mineral density in their dominant arm compared to national and regional players [55]. When the ranking position across various ages was compared, a better ranking position discriminated higher lean mass in boys Under 15 and Under 18, lower fat mass in boys Under 18, higher lean mass and bone density in girls Under 13, and greater upper arm girth in girls Under 11 and Under 13 [56]. Additionally, endomorph somatotype was highly associated with worse ranking positions in boys Under 18, while more successful players demonstrated an ectomorph somatotype [56].

#### 3.4.4. Sport

Nine studies compared anthropometric characteristics among athletes involved in different sports, including table tennis. A large cohort of athletes from 45 different sports revealed that players engaged in table tennis, hockey, handball, indoor soccer, rowing, and scuba diving had the highest fat percentage and skinfold measurements [50]. Similarly, table tennis players exhibited higher values in subscapular skinfold, sum of three skinfolds, and body fat percentage than sprinters [57]. A comparison between martial and non-martial art athletes highlighted a lower upper arm and calf circumference for table tennis players than judo and rugby players [51]. Moreover, table tennis players demonstrated a reduced maximal forearm and mid-thigh circumference compared to judo athletes, and a lower muscle mass than rugby, judo, and handball athletes [51]. Only one study examined body composition of female athletes involved in table tennis, weightlifting, football, and a control subgroup, revealing a lower fat-free mass percentage and water content for table tennis players only than controls [52]. Studies investigating young athletes (>18 years old) revealed a lower BCMI for junior elite male table tennis players than peers competing in badminton and lower BCMI variability across a wide BMI range compared to football and hockey players [49]. Regarding body fat, table tennis players exhibited lower levels compared with an age-matched male control group with no athletic background, but higher values than both football and hockey players [49]. Table tennis players demonstrated higher bone development compared to physically active children not involved in a specific sport [53], whilst a shorter leg length than other youth athletes (i.e., basketball, fencing, judo, swimming, and volleyball) was revealed [61]. None of the anthropometric measurements could discriminate table tennis players from the rest of Under 18 athletes from badminton, basketball, gymnastics, handball, judo, soccer, triathlon, and volleyball [59]. Finally, no significant differences emerged for the somatotype analysis among racket sports players (i.e., table tennis, badminton, and tennis) [54].

### 3.5. Determination of the Motor Profile

The outcomes evaluated in the included studies were compared according to age, gender, competitive level, and sport for the determination of the motor profile of table tennis players.

#### 3.5.1. Age

Four studies explored differences among age groups in table tennis players for the motor profile. Considering six age groups (i.e., Under 12, Under 14, Under 16, Under 20, and Senior [20–30 years]) and comparing each one with the subsequent older group, leg explosive and elastic strength increased with age in table tennis players until 20 years of age [41]. The original “Older” category (>30 years) from this study was excluded from our synthesis to prevent potential overlap with our upper age limit (40 years). Moreover, comparing top-level players aged 9 to 13 years, older players demonstrated higher scores [45]. This trend was consistent for the forearm isometric strength [41,45]. A similar pattern emerged for players from Under 11 to Under 18, where both genders exhibited a constant and linear improvement in explosive strength [43]. The enhancement in performance was more evident in girls until 15 years old, although they demonstrated a drop in performance in the Junior category. Regarding COD ability and speed performance, improvements were observed until the age of 20 years, followed by a decline [41]. Similar improvements were observed in the speed test among players aged 9–13 years [45]. Higher results were detected in the three tests for the TTSBT in players at the specialized training stage (13–17 years old players) compared to those in the targeted training stage (9–12 years old players) [60].

#### 3.5.2. Gender

Seven studies explored gender differences among table tennis players for the motor profile. Considering reaction ability, adult female players exhibited superior VRT than their male counterparts [44]. However, another study found no differences in VRT between males and females, except for seniors, where males outperformed females, and U11, where females achieved better results than males [35]. Regarding strength ability, male players demonstrated higher values in explosive [42,43,44] and elastic strength [42,43,44,58] than females. Specifically, in one study, young males obtained superior values in both SJ and CMJ tests starting from the cadet category (13–14 years old), with significant differences occurring in the junior category (15–17 years old) [43]. Moreover, adult males were superior to females in HANDG [44], while youth males performed better in the medicine ball throw test [45]. One study reported higher flexibility in females compared to males [61]. Concerning speed ability, males outscored their female counterparts in each age category from U11 to U18 for running speed [45], lateral displacement speed, and acceleration [35,44]. Finally, no significant differences in the three motor tests of the TTSBT were registered between young boys and girls [60].

#### 3.5.3. Competitive Level

Three studies compared the motor profile among different competitive levels of table tennis players. A positive correlation was found between performance in RT and ranking position in males Under 18 and Under 11, and in females Under 11 [34]. Moreover, Elite players outperformed state-level players in VRT tasks, COD ability, and explosive leg strength [47]. In addition, movement time (MT) was correlated with ranking in senior males [35]. In contrast, no significant differences between elite and regional table tennis players emerged in simple visual RT [40].

#### 3.5.4. Sport

Fifteen studies compared the motor profile of table tennis players with athletes from various sports or no-sport control groups. Regarding reaction ability, contrasting results emerged from several studies. Table tennis players demonstrated better performance in VRT than tennis players [31,34] and age-matched controls [34,36,40]. Similarly, table tennis players exhibited faster mean ART compared to footballers and healthy controls, and they showed better results in left-hand ART than tennis players [32,36]. Conversely, no differences were found among table tennis players and athletes of several sports in both VRT and ART [39,57]. The investigation of coincidence-anticipation timing accuracy demonstrated that table tennis players performed significantly better than tennis and badminton players at high stimulus velocity, while showing worse accuracy than badminton players at moderate velocity and tennis players at low velocity [31,33]. Considering strength qualities, Under 16 boys’ table tennis players exhibited greater dynamic back strength than age-matched basketball, fencing, judo, swimming, and volleyball athletes [61]. Similarly, 10–11-year-old players demonstrated superior upper limb strength than physically active peers not involved in a regular sport discipline [53]. Conversely, students participating in table tennis showed lower explosive leg strength than badminton and tennis players [38,48], and lower abdominal strength than badminton players [38]. Similarly, adult table tennis players showed lower values in reactive strength than basketball, badminton and tennis players [38,39]. Regarding COD ability, table tennis players outscored badminton and squash players [46], but their results were lower than handball players [39]. Primary school children significantly outscored the low-performance table tennis group in COD and speed tests, but not the high-performance group [37]. Moreover, both low- and high-performance table tennis groups outperformed their peers in the perceptuomotor skills [37].

Concerning motor coordination ability, table tennis players demonstrated better results compared to athletes involved in other sports (i.e., badminton, basketball, gymnastics, handball, judo, soccer, triathlon, and volleyball) [59].

### 3.6. Risk of Bias Assessment

Supplementary Table S4 reported the study quality and risk of bias of the included studies. Among the 31 included studies, 10 resulted in “high” quality, 13 were rated as “moderate” quality, and 8 were considered “low” quality. Most of the studies revealed limitations such as a lack of clear definitions for inclusion criteria, insufficient details in describing study subjects and settings, and inadequate strategies to deal with confounding factors.

## 4. Discussion

This systematic literature review aimed to provide a comprehensive synthesis of the anthropometric and motor characteristics of table tennis players, highlighting distinct characteristics across various age groups, gender, competitive levels, and different sports. The available evidence supports the identification of anthropometric and motor profiles for table tennis players and highlights some limitations which deserve the design of further studies tailored to the specific characteristics of table tennis performance. The identified characteristics provide a descriptive profile of table tennis players and may inform future longitudinal research investigating their potential relevance for talent identification and development.

The anthropometric profile of table tennis players varies significantly depending on gender and age, demonstrating age-related increases in breadths (humerus and femur), subscapular skinfold, and various girths [56,58]. Moreover, adult males predominantly exhibit an endomorph–mesomorph somatotype, while females show a prevalence of an endomorph–ectomorph profile [56,58,60]. While one specific subgroup analysis suggested a potential association between greater ectomorph somatotype and higher rankings [56], this cannot be generalized to the entire elite table tennis population. Furthermore, males also generally exhibit greater lean and muscle mass, particularly in the arms and legs, whilst females have higher fat mass and body fat percentage [55,56,58]. These observations are also aligned with well-established gender differences in body composition within the general population [76]. The analysis of competitive level demonstrated higher lean mass in high-competitive young and senior players, while high-competitive female players have higher bone mineral density [55,56]. The comparisons among different sports revealed that table tennis players have lower limb circumferences and lower lean and muscle mass compared to specific strength- and power-based sports (i.e., rugby and judo) [51], although no difference in fat-free mass was observed between table tennis and weightlifters [52]. Conversely, adult table tennis players show higher skinfolds and body fat levels compared to speed- and agility-based athletes like sprinters, hockey, and football players [49,52]. However, these findings should be interpreted with caution, considering the low-quality assessment of the related studies [51,52], and, therefore, rigorous study designs and methodology are required to fully support these conclusions. Finally, the absence of significant differences in the somatotype variables among table tennis, badminton, and tennis players suggests comparable morphological characteristics within these racket sports [54].

These findings reflect the specificity of table tennis demands, characterized by rapid movements and fine motor coordination, which favor athletes with distinct anthropometric characteristics, body compositions, and somatotypes. Specifically, table tennis players with leaner physiques may demonstrate an advantage in executing rapid multidirectional movement patterns, dynamic strokes, and quick recovery movements, crucial to gaining a tactical advantage during table tennis matches.

In the determination of the motor profile of table tennis players, particular attention has been given to the reactive capabilities. However, when evaluating the results, it is crucial to distinguish among simple laboratory RT, MT, coincidence-anticipation timing, and table tennis-specific reactive performance. Indeed, evidence reveals better simple RT and faster MT for higher-ranked table tennis players [35,47]. Moreover, some studies report that table tennis players generally have a faster laboratory RT than tennis players, footballers, and age-matched controls [31,32,34,36,40], while others found no significant differences among different sports (e.g., fencing, runners, and basketball and handball players) or control groups [39,57]. Female table tennis players often show superior VRT than males; however, age-dependent disparities emerged, with males generally outperforming females in senior categories, whereas females excel in the U11 category [34,44]. Importantly, faster RT in a laboratory setting does not, by itself, establish enhanced cognitive processing or sport-specific responsiveness during a match. Regarding coincidence-anticipation timing, contradictory findings emerge depending on stimulus velocities. Indeed, table tennis players demonstrate superior accuracy compared to tennis and badminton players under high stimulus velocity, but worse or similar accuracy at lower or medium velocities [31,33]. The superior performance of table tennis players in RT and fast coincident-anticipation tasks could be driven by the dimensions of the table and equipment materials (i.e., racket and ball). These factors affect ball speed and trajectory, and consequently, the time available for the players to react and anticipate opponents’ shots. However, unless directly tested in sport-specific conditions, this remains a hypothesis. These discrepancies in the literature strongly emphasize the need for a valid and reliable reactive testing protocol tailored to the demands of the sport, essential for an accurate assessment of the ability of processing speed, reaction, and quickness of table tennis players [77]. Moreover, considering the inclusion of studies with low-quality assessments [31,34], more research with more precise participant selection criteria and rigorous methodology is needed to confirm these observations.

The evaluation of strength qualities highlights that high-level players achieve better results in horizontal jumps [47]. However, further investigations are required to confirm these findings. Youth table tennis players outperform athletes from basketball, fencing, judo, swimming, and volleyball only in dynamic back strength [61], and physically active controls in handgrip strength [53]. Conversely, table tennis players show lower reactive and explosive strength compared to basketball, badminton, and tennis players assessed through vertical jump tests (i.e., squat jump and countermovement jump tests) [38,39,48]. Explosive and elastic strength of the lower limbs and forearm isometric strength increase with advancing age until 20 years old, with the same trend for both boys and girls, with a notable decline in junior (15–17 years old) girls’ performance [43,45]. Males exhibit greater values for explosive and elastic strength, with significant differences observed starting from the cadet category (13–14 years old) [42,43,44,45,58]. These findings confirm the gender disparities in jump performance identified by various authors across different sports [78]. Evidence highlights the limitations of applying conventional strength tests in table tennis, as vertical jumps or isometric measures may fail to capture the specific strength qualities characterizing the performance. Therefore, specific protocols mimicking table tennis movement patterns are essential for a more accurate evaluation of players’ strength profiles.

Higher COD ability is associated with better competitive levels. In fact, high-level players outperform their lower-level counterparts in general COD tasks [47]. Furthermore, primary school children excel in COD ability when compared to lower-performance table tennis players, but they do not outscore the higher-performance group [37]. This indicates that COD ability is significantly influenced by the competitive levels in table tennis, with the high-performance group exhibiting superior results, likely due to more advanced training and skill development. Table tennis players also exhibit a superior COD ability compared to players from racket sports (badminton and squash), but they demonstrate worse performance than handball players [39,46]. A similar trend was also observed in sprint performance [41,45]. Males outperform females in running speed and lateral displacement speed across all age categories, with faster MT contributing to the achievement of a greater performance [35,44,45]. Table tennis players also exhibit superior motor coordination abilities in specific tests like backward balance and sideways moving, which allowed to discriminate table tennis players and badminton, basketball, gymnastics, handball, judo, soccer, triathlon, and volleyball [59]. The enhanced COD ability and motor coordination observed in table tennis players support their ability to perform quick and diverse footwork actions and maintain balance while executing complex technical skills, highlighting the critical link between footwork and stroke effectiveness [79].

### Limitations

It is essential to acknowledge the limitations of the studies included in this review for a comprehensive understanding of the available evidence. Given the cross-sectional nature of the included studies, it must be emphasized that this profile provides a descriptive characterization of current performance status, and findings should not be strictly interpreted as predictive markers for future talent identification or selection. The included literature is further limited by small table tennis subsamples, inconsistent definitions of competitive levels, and lack of control for training age. Another major limitation across the studies evaluating youth athletes is the widespread absence of biological maturity assessments. Since anthropometric and physical performance outcomes in children and adolescents are strongly confounded by biological maturation, relying solely on chronological age limits the reliability of the comparisons within youth subgroups. A critical limitation is the variability in anthropometric and motor measurements, as well as the highly heterogeneous comparator sports, across the reviewed studies. This methodological discrepancy can affect the comparability of results and the generalizability of conclusions. Furthermore, the frequent absence of effect sizes, confidence intervals, or complete descriptive statistics in the included studies prevented the calculation of quantitative effect estimates, and the synthesis therefore relied on structured effect direction. Additionally, potential biases in study methodologies, such as differences in participant selection criteria and inclusion criteria, may impact the reliability and applicability of the results in practical sports settings. Moreover, it is essential to acknowledge that several studies included in this review have been assessed as “low” quality, and the robustness of conclusions may be impacted by the quality of the studies. Finally, the restriction to English-language full texts should be acknowledged as a potential source of language bias, which may have excluded relevant data.

Further investigations are needed to understand how specific anthropometric and motor characteristics could influence playing styles in table tennis. Studies focusing on these interactions could provide more detailed insights into optimizing motor characteristics to enhance sports performance. Furthermore, due to the lack of studies, it is crucial to confirm the observed trends with studies that include larger and more diverse samples of table tennis players across different competitive levels. This would help to strengthen existing evidence and improve overall understanding of anthropometric and motor characteristics in table tennis.

## 5. Conclusions

The results of the current review highlight the distinct anthropometric and motor characteristics of table tennis players, which are hypothesized to be shaped by the unique demands of the sport. Anthropometrically, adult male players predominantly exhibit an endomorph–mesomorph somatotype with greater lean mass and muscle girth, whereas females are characterized by an endomorph–ectomorph profile and higher fat mass. Notably, higher-ranked athletes generally display greater lean mass. Compared to athletes involved in specific strength-based sports, table tennis players tend to have lower limb circumferences and lower lean and muscle mass. Conversely, they exhibit higher body fat percentages and subscapular skinfold measurements compared to specific speed- and agility-based sports. The motor profile of table tennis players seems to be characterized by highly developed reaction and movement times in higher-ranked players, as well as superior change-of-direction abilities. When comparing table tennis players to athletes from other racket sports, they tend to exhibit superior coincidence-anticipation timing specifically under high stimulus velocities, alongside distinctive specific motor coordination abilities. In terms of strength, table tennis players show distinct proficiency in horizontal jumps and dynamic back strength, despite lower vertical explosive power compared to other racket sports. Given the cross-sectional nature of the available evidence, the identified characteristics should be interpreted as descriptive features of table tennis players rather than as predictive markers for talent identification. Future longitudinal studies are required to establish their potential predictive value, employing larger and more diverse samples, and homogeneous and sport-specific testing protocols to effectively evaluate table tennis performance.

## Data Availability

The original contributions presented in this study are included in the article/Supplementary Material. Further inquiries can be directed to the corresponding author(s).

## Supplementary Materials

The following supporting information can be downloaded at https://www.mdpi.com/article/10.3390/jfmk11030334/s1: Supplementary File S1: PRISMA 2020 checklist; Table S1: Search strings utilized across the databases and platforms; Table S2: List of excluded full-text articles; Table S3: Studies characteristics and main findings; Table S4: Quality assessment of the included studies, according to the Joanna Briggs Institute (JBI) Critical Appraisal Checklist for cross-sectional studies.

## Abbreviations

The following abbreviations are used in this manuscript:

TT	Table tennis
%BF	Body fat percentage
FM	Fat mass
FFM	Fat-free mass
FFSTM	Fat-free soft tissue mass
BMD	Bone mineral density
BMC	Bone mineral content
BCMI	Body cell mass index
CSA	Cross-sectional area
RT	Reaction time
VRT	Visual reaction time
ART	Auditory reaction time
MT	Movement time
SJ	Squat jump
CMJ	Countermovement jump
HANDG	Handgrip test
COD	Change-of-direction
SAR	Sit and reach test
TTSBT	Table Tennis Specific Battery Test
