# Supplementary material for: Anthropometric and Motor Profile of Table Tennis Players: A Systematic Literature Review
Source: J Funct Morphol Kinesiol. 2026 Aug 26;11(3):334. doi: 10.3390/jfmk11030334 (PMC13608705; doi:10.3390/jfmk11030334)
Supplement: Supplementary file 1 [file jfmk-11-00334-s001.zip › Supplementary Table S1_Guarnieri et al..pdf]

Table S1. Search strings utilized across the databases and platforms.

Database	Search String 1 (Anthropometric Profile)	Search String 2 (Motor Profile)
PubMed	("anthropometry"[Title/Abstract] OR "anthropometric"[Title/Abstract] OR "somatic"[Title/Abstract] OR "somatotype"[Title/Abstract] OR "body composition"[Title/Abstract]) AND "table tennis"[Title/Abstract]	("motor task"[Title/Abstract] OR "motor level"[Title/Abstract] OR "motor performance"[Title/Abstract] OR "motor fitness"[Title/Abstract] OR "physical performance"[Title/Abstract] OR "physical fitness"[Title/Abstract] OR "physical level"[Title/Abstract]) AND "table tennis"[Title/Abstract]
Scopus	TITLE-ABS-KEY ((anthropometry OR anthropometric OR somatic OR somatotype OR "body composition") AND "tab<br>le tennis")	TITLE-ABS-KEY (("motor task" OR "motor level" OR "motor performance" OR "motor fitness" OR "physical performance" OR "physical fitness" OR "physical level") AND "table tennis”)
Web of Science	TS=((anthropometry OR anthropometric OR somatic OR somatotype OR "body composition") AND "table tennis")	TS=(("motor task" OR "motor level" OR "motor performance" OR "motor fitness" OR "physical performance" OR "physical fitness" OR "physical level") AND "table tennis")
Science Direct	(anthropometry OR anthropometric OR somatic OR somatotype OR "body composition") AND "table tennis"	("motor task" OR "motor level" OR "motor performance" OR "motor fitness" OR "physical performance" OR "physical fitness" OR "physical level") AND "table tennis"
BASE	(anthropometry OR anthropometric OR somatic OR somatotype OR "body composition") AND "table tennis"	("motor task" OR "motor level" OR "motor performance" OR "motor fitness" OR "physical performance" OR "physical fitness" OR "physical level") AND "table tennis"
Semantic Scholar	(anthropometry OR anthropometric OR somatic OR somatotype OR "body composition") AND "table tennis"	("motor task" OR "motor level" OR "motor performance" OR "motor fitness" OR "physical performance" OR "physical fitness" OR "physical level") AND "table tennis"
