# Supplementary material for: Anthropometric and Motor Profile of Table Tennis Players: A Systematic Literature Review
Source: J Funct Morphol Kinesiol. 2026 Aug 26;11(3):334. doi: 10.3390/jfmk11030334 (PMC13608705; doi:10.3390/jfmk11030334)
Supplement: Supplementary file 1 [file jfmk-11-00334-s001.zip › Supplementary Table S2_Guarnieri et al.pdf]

Table S2. List of excluded full-text articles.

Athors, Year	Article	Reason
Abokyi et al., 2021	Differential effect of maximal incremental treadmill exercise on tear secretion and tear film stability in athletes and non-athletes.	Outcome not relevant
Assar et al., 2022	The Relationship Between Reaction Time, Eye-Hand Coordination with Visual Field in Elite Tennis Players.	Outcome not relevant
Bańkosz, 2012	The kinesthetic differentiation ability of table tennis players.	Outcome not relevant
Bańkosz and Skarul, 2010	Changes in the level of kinesthetic differentiation ability in table tennis players.	Outcome not relevant
Bastik et al., 2012	Investigation of basic motor skills according to TGMD-2 test on male athletes of 10 ages group who participated to competitions in different sports branches.	Outcome not relevant
Behdari et al., 2016	Anthropometric Characteristics, Body Composition and Somatotype of 9-12 Years Old Table Tennis Players.	Full text not in English
Cherif et al., 2022	Anthropometry, body composition, and athletic performance in specific field tests in Paralympic athletes with different disabilities.	Subject with disability
Deprà et al., 2022	Effects of table tennis practice on balance and physical fitness in the elderly.	Out of range age
Doherty et al., 2018	Determinants for table tennis performance in elite Scottish youth players using a multidimensional approach: A pilot study.	Not cross-sectional
Durán-Agüero et al., 2016	Anthropometric profile of elite Chilean Paralympic athletes.	Subject with disability
Gonzàlez-Garcìa et al., 2019	Differences in anger depending on sport performance in table tennis players.	Outcome not relevant
Hung et al., 2004	Assessment of reactive motor performance with event-related brain potentials - Attention processes in elite table tennis players.	Outcome not relevant
Lenoir et al., 2009	Saccadic eye movements and finger reaction times of table tennis players of different levels.	Outcome not relevant
Michalski et al., 2017	The Anaerobic Power of Women who have Undergone Physical Training.	Outcome not relevant
Martin et al., 2015	Influence of the playing style on the physiological responses of offensive players in table tennis.	Outcome not relevant
Muñoz et al., 2021	Somatotype and polymorphism of the ACTN3 and ACE gene in Chilean table tennis players.	Full text not in English
Naderi et al., 2018	A retrospective comparison of physical health in regular recreational table tennis participants and sedentary elderly men.	Out of range age
Naderi et al., 2018	Body composition and lipid profile of regular recreational table tennis participants: a cross-sectional study of older adult men.	Out of range age
Nikolić et al., 2014	The relationship between reaction time, eye-hand coordination with visual field in elite table tennis player.	Outcome not relevant
Picabea et al., 2017	Physical fitness analysis in male and female table tennis players and their relationship to competition performance.	Full text not in English
Pluta et al., 2020	The Motor and Leisure Time Conditioning of Young Table Tennis Players' Physical Fitness.	Outcome not relevant
Pradas et al., 2013	Anthropometric Characteristics, Body Composition and Somatotype of High Level Table Tennis Players.	Full text not in English
Pradas et al., 2007	Anthropometric profile, somatotype, and body composition of young table tennis players.	Full text not in English
Rana and Rajpoot, 2013	Impact and Role of Selected Coordinative Abilities in Racket Sports.	Not cross-sectional
Rodrigues et al., 2002	Head, eye and arm coordination in table tennis.	Outcome not relevant

Saiti et al., 2014	Asymmetry of anthropometric dimensions and motor efficiency of table tennis players from Macedonia competing in the super and the first league.	Outcome not relevant
Smajić et al., 2017	Differences of morphological characteristics and motor abilities between cadet table tennis players and children of pupils non-athletes.	Full text not in English
Yao and Chao, 2022	Physical Fitness Training and Reaction Speed In Sports Table Tennis Players.	Not cross-sectional
Yáñez Sepúlveda et al, 2015	Anthropometric Chilean Table Tennis Players of Competitive Features.	Full text not in English
Yue et al., 2025	Psychological characteristics of the national Hungarian table tennis team.	Outcome not relevant
Van Biesen et al., 2012	Technical proficiency among table tennis players with and without intellectual disabilities.	Subject with disability
Veita et al., 2018	Anthropometrical characteristics of Cuban sporting population: Reference data from a high performance national teams, 1992–2014.	Not cross-sectional
Zagatto et al., 2014	Analysis of cardiopulmonary and metabolic variables measured during laboratory and sport-specific incremental tests for table tennis performance prediction.	Outcome not relevant
