# Supplementary material for: Anthropometric and Motor Profile of Table Tennis Players: A Systematic Literature Review
Source: J Funct Morphol Kinesiol. 2026 Aug 26;11(3):334. doi: 10.3390/jfmk11030334 (PMC13608705; doi:10.3390/jfmk11030334)
Supplement: Supplementary file 1 [file jfmk-11-00334-s001.zip › Supplementary Table S3_Guarnieri et al.pdf]

Table S3. Studies characteristics and main findings.

Study, Year; Country	Age (range; mean ± SD)	Sample Size	Sport	Groups Comparison	Measurements	Main findings
Ak and Koçac, 2010 [31]; Turkey	12.3 ± 1.1	N = 208<br>M = 111; F = 97	Table tennis = 101<br>Tennis = 107	Sport	Motor: VRT, coincidence-anticipation timing accuracy.	Table tennis players showed faster VRT but worse coincidence-anticipation timing accuracy than tennis players.
Akhani et al., 2015 [32]; India	18.5 ± 0.5	N = 90<br>M = 90	Table tennis = 30<br>Football = 30<br>Control = 30	Sport	Motor: ART.	Table tennis players showed faster mean ART than footballers and healthy controls.
Akpinar et al., 2012 [33]; Turkey	10-15	N = 90<br>M=45; F=45	Table tennis = 30<br>Badminton = 30<br>Tennis = 30	Sport	Motor: coincidence-anticipation timing accuracy.	Table tennis players outperformed tennis and badminton players under high stimulus velocity, but they exhibited worse performance compared to tennis players under low stimulus velocity and to badminton players under moderate stimulus velocity.
Alhowikan et al., 2022 [57]; Saudi Arabia	18-26	N = 58	Table tennis = 12<br>Fencing = 30<br>Long distance runner = 13<br>Sprinter = 12<br>Control = 8	Sport	Anthropometric: body mass, height, body max index, body fat percent, skinfold thicknesses.<br>Motor: VRT, ART, lower body strength.	Table tennis players showed higher values in subscapular skinfold, sum of skinfolds, and body fat percentage than sprinters.
Bandyopadhyay et al., 2019 [49]; India	16.7 ± 2.3	N = 414<br>M = 414	Table tennis = 74<br>Badminton = 63<br>Football = 95<br>Hockey = 110<br>Control = 72	Sport	Anthropometric: body mass, height, body composition.	Table tennis payers showed: lower values of body cell mass percentage than hockey, football, and badminton players; lower values of body cell max index than badminton players; lower values of body fat than controls; higher values of body fat than football and hockey players.
Can et al., 2014 [34]; Turkey	10-12;<br>12.00 ± 0.8	N = 51<br>M = 51	Table tennis = 18<br>Tennis = 17<br>Control = 16	Sport	Motor: simple ART and VRT.	Table tennis players outperformed tennis players and controls in right hand VRT and tennis players in left hand ART.
Castellar et al., 2019 [35]; Spain	12.7 ± 4.3	N = 116<br>M = 71; F = 45	Table tennis = 116	Gender; Competitive level	Motor: VRT and lateral displacement.	Males U18, U15, U13 and U11 outscored their female's counterparts in total time of response, due to a faster MT observed in males. Both lower VRT and MT seem to be key variables of performance in table tennis

Chattarjee et al., 2016 [58]; India	10-20	N = 29<br>M = 14; F = 15	Table tennis = 29	Gender	Anthropometric: body mass, height, eight skinfold thicknesses, four girths, three breadths, body composition, somatotype.<br>Motor: elastic leg strength, handgrip strength, flexibility, COD ability.	Endomorph-mesomorph somatotype was revealed in males and endomorph-ectomorph somatotype in females. Females showed higher body fat percentage and total fat content; conversely, males showed higher lean body mass, biepicondylar humerus and femur breadths. Males outscored females in elastic leg strength.
Deepa and Sirdesai, 2016 [36]; India	18-30	N = 60<br>M = 60	Table tennis = 30<br>Control = 30	Sport	Motor: VRT, ART.	Table tennis players outscored controls in ART and VRT for red, green, and blue color.
Faber et al., 2017 [37]; The Netherlands	8-10	N = 267<br>M = 162; F = 105	Table tennis = 146<br>Control = 121	Competitive level; Sport	Motor: perceptuo-motor skills (sprint, COD ability, vertical jumps, speed while dribbling, aiming the target, balls skills, throwing a ball, eye-hand coordination).	Both the low- and high-performance table tennis groups outperformed their peers from the primary school in “ball control” tests. The primary school children significantly outscored low-performance group in sprint and COD ability test.
Garrido-Chamorro et al., 2012 [50]; Spain	21.3 ± 6.1	N = 2500<br>M = 1771; F = 729	Table tennis = 21<br>Other sports = 2479	Sport	Anthropometric: six skinfolds thickness, body composition.	Individuals practicing disciplines such as table tennis, hockey, handball, indoor soccer, rowing, and scuba diving had the highest fat percentages and thereby the highest values for six sum of skinfolds.
Ghosh and Mondal, 2016 [38]; India	14-16<br>15.2 ± 0.5	N = 60<br>M = 60	Table tennis = 30<br>Badminton = 30	Sport	Motor: explosive strength, abdominal strength.	Table tennis players showed lower values in explosive and abdominal strength than badminton players
Horníková et al., 2023 [39]; Slovakia	20.0 ± 1.2	N = 33<br>M = 33	Table tennis = 15<br>Basketball = 11<br>Handball = 7	Sport	Motor: simple and choice VRT, VRT to slow and fast generating stimuli, explosive strength, elastic-explosive strength, reactive strength, COD ability, speed.	Table tennis players exhibited worse reactive strength than basketballers, and worse COD ability values compared to handball players.
Kim, 2016 [51]; Korea	20.2 ± 1.2	N = 120<br>M = 120	Table tennis = 20<br>Badminton = 20<br>Handball = 20<br>Judo = 20<br>Taekwondo = 20<br>Rugby = 20	Sport	Anthropometric: body mass, height, four skinfold thicknesses, four girths, muscle mass.	Table tennis players showed lower values in upper arm and calf circumferences than judo and rugby athletes, lower values in maximal forearm and mid-thigh circumference than judo, and lower values in muscle mass than rugby, judo, and handball athletes.

Padulo et al., 2016 [40]; Italy	27.4 ± 2.5	N = 28<br>M = 28	Table tennis = 17<br>Control = 11	Competitive level	<u>Motor</u>: VRT, response time, playing ability.	Table tennis players outscored controls in VRT. Elite outperformed regionals players in the playing ability test.
Picabea et al., 2021 [41]; Spain	19.7 ± 11.2	N = 71<br>M = 61; F = 10	Table tennis = 71	Age	<u>Motor</u>: handgrip strength, elastic leg strength, flexibility, COD ability, speed.	Forearm isometric strength improved with age. Elastic leg strength, COD ability and sprint improved until 20 years of age and thereafter deteriorate.
Picabea and Ozaeta-Beaskoetxea; 2025 [42]; Spain	16-18<br>16.9 ± 0.8	N = 40<br>M = 20; F = 20	Table tennis = 40	Gender	<u>Motor:</u> explosive leg strength, elastic leg strength.	Male players achieved higher results in all strength tests.
Pilis et al., 2019 [52]; Poland	18-25<br>21.5 ± 3.5	N = 51<br>F = 51	Table tennis = 12<br>Football = 15<br>Weightlifting = 12<br>Control = 12	Sport	<u>Anthropometric</u>: body mass, height, body composition.	Table tennis players showed greater body mass index, lower fat-free mass percentage and lower content of water percentage than controls.
Pion et al., 2015 [59]; Belgium	16.1 ± 0.8	N = 141<br>M = 141	Table tennis = 12<br>Badminton = 12<br>Basketball = 27<br>Gymnastic = 8<br>Handball = 28<br>Judo = 8<br>Triathlon = 12<br>Volleyball = 20	Sport	<u>Anthropometric</u>: body mass, height, sitting height, body composition.<br><u>Motor</u>: explosive leg strength, upper body strength, flexibility, COD ability, speed, gross motor coordination, dribbling performance, overarm throwing skills.	Gross motor coordination tests were the only measurement that discriminated table tennis and non-table tennis players.
Pluta et al., 2021 [60]; Poland	13.4 ± 1.7	N = 87<br>M = 49; F = 38	Table tennis = 87	Age; Gender	<u>Anthropometric</u>: body mass, height, sitting height, arm span, five skinfold thicknesses, five girths, two breadths, arm span, somatotype.<br><u>Motor</u>: RT and displacement speeds during the game.	Body weight, height and sitting height, humerus and femur diameter, subscapular skinfold, arm span, and biceps, waist, hip, thigh, and calf girth increased with increasing age. Somatotype was predominantly mesomorph in boys and ectomorph in girls. Height, weight, body mass index, humerus and femur diameter, biceps, calf, and waist girth were greater in males. Older player obtained better results in all specific motor test.
Pradas et al., 2013 [43]; Spain	7-17	N = 165<br>M = 95; F = 70	Table tennis = 165	Age; Gender	<u>Motor</u>: explosive leg strength, elastic leg strength.	Males registered a significant improvement in junior players for both explosive and elastic leg strength tests, while females exhibited a significant improvement from U11 to the cadet

						female categories, except for junior players. Males outscored females starting from Junior category in both tests.
Pradas et al., 2021 [53]; Spain	10-11	N = 374<br>M = 182; F = 192	Table tennis = 182<br>Control = 192	Sport	Anthropometric: body mass, height, eight skinfold thicknesses, four girths, three breadths, body composition, somatotype.<br>Motor: hand grip strength, flexibility.	Table tennis players aged 10–11 years presented higher bone development and hand grip strength than controls.
Pradas et al., 2021 [56]; Spain	7-39	N = 495<br>M = 288; F = 207	Table tennis = 495	Age;<br>Gender;<br>Competitive level	Anthropometric: body mass, height, eight skinfold thicknesses, four girths, three breadths, body composition, somatotype, cross sectional muscle area.	Somatotype was predominantly mesomorph in men and endomorph in women. Better ranking positions were associated with higher lean mass in boys U15 and U18, lower fat mass in boys U18, greater upper arm girth, lean mass and bone density in girls U13. Endomorph somatotypes were negatively related to performance in boys U18, while ectomorph profiles seems more effective.
Pradas et al., 2022 [44]; Spain	10-38	N = 48<br>M = 24; F = 24	Table tennis = 48	Gender	Motor: RT and lateral displacements, explosive leg strength, elastic leg strength, handgrip strength.	Males outscored females in hand grip strength, vertical jumps (SJ and CMJ), lateral displacement and acceleration. Female players showed a faster RT.
Pradas et al., 2023 [45]; Spain	9-13	N = 352<br>M = 203; F = 149	Table tennis = 352	Age;<br>Gender	Motor: explosive leg strength, elastic leg strength, handgrip strength, flexibility, speed.	Older players obtain better results in strength and speed tests. Females obtained higher values in flexibility, while males performed better in strength tests (medicine ball throwing) and running speed.
Singh et al., 2017 [46]; India	16-19	N = 150<br>M = 150	Table tennis = 50<br>Badminton = 50<br>Squash = 50	Sport	Motor: COD ability, speed.	Table tennis players outperformed badminton and squash players in COD ability.
Srinet and Mishra, 2025 [47]; India	15-25	N=180<br>M=180	Table tennis = 180	Competitive Level	Motor: VRT, explosive leg strength, handgrip strength, upper body strength, COD ability, speed.	Elite players outperformed State level players in VRT, COD ability, and explosive leg strength.
Varmiş et al., 2024 [54]; Turkey	16-18	N = 36<br>M = 20; F = 16	Table tennis = 12<br>Badminton = 15<br>Tennis = 9	Sport	Anthropometric: body mass, height, body composition, somatotype.	No significant differences emerged in any of the investigated variables.

Wang et al., 2024 [48]; China	21 ± 2	N = 60<br>M = 60	Table tennis = 20<br>Badminton = 20<br>Tennis = 20	Sport	Motor: explosive leg strength, elastic leg strength.	Table tennis players showed lower results in both strength tests than badminton players.
Zagatto et al., 2015 [55]; Brazil	24 ± 7	N = 64<br>M = 45; F = 19	Table tennis = 64	Competitive level; Gender	Anthropometric: body mass, height, body composition.	Male players presented higher FFM, and lower FM and %BF than females in all competitive levels. International male players exhibited lower FFM and FFSTM in their dominant arm than national players, and in their non-dominant arm than both national and regional players. International female players showed higher BMD than national and regional players specifically in their dominant arm.
Zhao et al., 2019 [61]; China	14-16	N = 97<br>M = 97	Table tennis = 15<br>Basketball = 7<br>Fencing = 20<br>Judo = 20<br>Swimming = 10	Sport	Anthropometric: body mass, height, sitting height, arm span, two skinfold thicknesses, three girths, three lengths, two widths, two circumferences, Achilles tendon length, subscapular angle. Motor: simple RT, dynamic back strength.	Table tennis players showed shorter lower leg length than other athletes. Dynamic back strength is greater in table tennis players compared to the athletes from other sports.

Abbreviations: ART = auditory reaction time; BMD = bone mineral density; %BF = body fat percentage; CMJ = countermovement jump; COD = change of direction; FM = fat mass; FFM = fat-free mass; FFSTM = fat-free soft tissue mass; MT = movement time; RT = reaction time; SJ = squat jump; VRT = visual reaction time
