# Supplementary material for: Anthropometric and Motor Profile of Table Tennis Players: A Systematic Literature Review
Source: J Funct Morphol Kinesiol. 2026 Aug 26;11(3):334. doi: 10.3390/jfmk11030334 (PMC13608705; doi:10.3390/jfmk11030334)
Supplement: Supplementary file 1 [file jfmk-11-00334-s001.zip › Supplementary Table S4_Guarnieri et al.pdf]

Table S4. Quality assessment of the included studies, according to the Joanna Briggs Institute (JBI) Critical Appraisal Checklist for cross-sectional studies.

	Were the criteria for inclusion in the sample clearly defined?	Were the study subjects and the setting described in detail?	Was the exposure measured in a valid and reliable way?	Were objective, standard criteria used for measurement of the condition?	Were confounding factors identified?	Were strategies to deal with confounding factors stated?	Were the outcomes measured in a valid and reliable way?	Was appropriate statistical analysis used?	**Total score**	**Quality**
AK and Koçac, (2010) [31]	0	0	NA	0	0	0	1	0	**1**	**Low**
Akhani et al., (2015) [32]	1	1	NA	1	0	0	0	1	**4**	**Medium**
Akpinar et al., (2012) [33]	1	0	NA	1	1	1	1	1	**6**	**High**
Alhowikan et al., (2022) [57]	0	0	NA	1	1	0	1	1	**4**	**Medium**
Bandyopadhyay et al., (2019) [49]	0	0	NA	1	1	0	1	1	**4**	**Medium**
Can et al., (2014) [34]	0	0	NA	1	0	0	1	1	**3**	**Low**
Castellar et al., (2019) [35]	0	0	NA	1	1	1	1	1	**5**	**Medium**
Chattarjee et al., (2016) [58]	0	1	NA	1	0	0	0	1	**3**	**Low**
Deepa and Sirdesai, (2016) [36]	1	0	NA	1	1	0	0	1	**4**	**Medium**
Faber et al., (2017) [37]	0	1	NA	1	1	1	1	1	**6**	**High**
Garrido-Chamorro et al., (2012) [50]	0	1	NA	1	0	0	1	0	**3**	**Low**
Ghosh and Mondal, (2016) [38]	0	0	NA	0	0	0	1	1	**2**	**Low**
Horníková et al., (2023) [39]	1	0	NA	1	0	0	1	1	**4**	**Medium**
Kim (2016) [51]	0	0	NA	0	0	0	1	1	**2**	**Low**
Padulo et al., (2016) [40]	1	1	NA	1	1	1	0	1	**6**	**High**
Picabea et al., (2021) [41]	1	1	NA	1	1	0	1	1	**6**	**High**
Picabea and Ozaeta-Beaskoetxea (2025) [42]	1	1	NA	1	1	0	1	1	**6**	**High**
Pilis et al., (2019) [52]	0	0	NA	1	0	0	1	1	**3**	**Low**
Pion et al., (2015) [59]	0	0	NA	1	1	1	1	1	**5**	**Medium**
Pluta et al., (2021) [60]	1	0	NA	1	1	0	1	1	**5**	**Medium**
Pradas et al., (2013) [43]	0	1	NA	1	1	1	1	1	**6**	**High**
Pradas et al., (2021) [53]	1	1	NA	1	1	1	1	1	**7**	**High**
Pradas et al., (2021) [56]	1	0	NA	1	1	0	1	1	**5**	**Medium**
Pradas et al., (2022) [44]	1	0	NA	1	1	0	1	1	**5**	**Medium**
Pradas et al., (2023) [45]	1	1	NA	1	1	1	1	1	**7**	**High**
Singh et al., (2017) [46]	0	0	NA	0	0	0	0	0	**0**	**Low**
Srinet and Mishra (2025) [47]	1	1	NA	1	0	0	0	1	**4**	**Medium**

Varmiş et al., (2024) [54]	1	0	NA	1	0	0	1	1	**4**	**Medium**
Wang et al., (2024) [48]	0	1	NA	0	1	0	1	1	**4**	**Medium**
Zagatto et al., (2015) [55]	0	1	NA	1	1	1	1	1	**6**	**High**
Zhao et al., (2019) [61]	0	1	NA	1	1	1	1	1	**6**	**High**

Abbreviations: NA = not applicable.
